# Case report: hibernoma, a rare male breast mass

**DOI:** 10.1093/jscr/rjae820

**Published:** 2024-12-26

**Authors:** Joanna Sajdlowska, Funmilayo Fawole, Anjeli Patel, Nawras Radwan, James Yang, Derick Christian

**Affiliations:** General Surgery Department, St. Joseph's University Medical Center, Paterson, NJ, United States; School of Medicine, St. George’s University, University Centre Grenada, West Indies, Grenada; University of New England College of Osteopathic Medicine, 11 Hills Beach Road, Biddeford, ME 04005, United States; School of Medicine, St. George’s University, University Centre Grenada, West Indies, Grenada; General Surgery Department, St. Joseph's University Medical Center, Paterson, NJ, United States; General Surgery Department, St. Joseph's University Medical Center, Paterson, NJ, United States

**Keywords:** hibernoma, breast mass, male breast mass, brown fat tumor

## Abstract

Hibernomas are rare, benign neoplasms characterized by the presence of brown adipose tissue. Although these tumors may arise in any region of brown fat, they predominantly occur in the thigh, shoulder, back, and neck. Hibernomas are rarely found in mammary tissue, with a higher prevalence in females than males. This case report highlights a unique presentation of a 20 cm hibernoma mass found in the right breast of a 31-year-old male. A diagnostic bilateral mammogram, unilateral MRI, and ultrasound were used to evaluate the mass. The patient underwent an intra-operative ultrasound-guided needle biopsy and left total mastectomy, revealing the diagnosis of hibernoma with fat necrosis. This report aims to delineate the pathological, diagnostic, and clinical features associated with breast hibernomas and to offer a comprehensive review of the literature on the subject. This case report also serves to expand the differential for breast mass in male patients, with a focused aim to prevent delayed diagnosis and treatment.

## Introduction

Hibernomas are rare, benign tumors characterized by brown adipose tissue. Typically, these tumors exhibit slow growth and are asymptomatic, often presenting in the thigh, upper torso, or neck subcutaneous tissues [1]. The rarity of hibernomas is attributed to their origin from fetal brown fat, which is infrequently retained in adulthood. Fewer than 200 cases of hibernoma have been documented in the literature [1]. This case report highlights an unusual presentation of a hibernoma located in the mammary tissue of a 31-year-old male patient and serves to expand the differential for breast masses in male patients.

## Case report

A 31-year-old male with a past medical history of asthma and obesity was referred to the surgical oncology clinic because of a left breast mass. The mass was first noticed approximately 3 years ago which had been progressively enlarging and increasingly painful. The patient was initially in 2021 but was lost to follow-up due to limited access to medical care.

On presentation, he was hemodynamically stable. As compared to a normal appearing right male breast, the left breast had a large, soft, uniform palpable mass. The skin appeared to have enlarged veins, and the nipple was inferiorly deviated with no signs of discharge. There was no evidence of skin discoloration, dimpling, or thickening. The remaining systems were unremarkable; he denied abnormal sexual features and function, trauma or injuries to the left breast, and denied a family history of breast disorders or breast cancer. All laboratory values, including tumor markers, were within normal limits.

**Figure 1:**
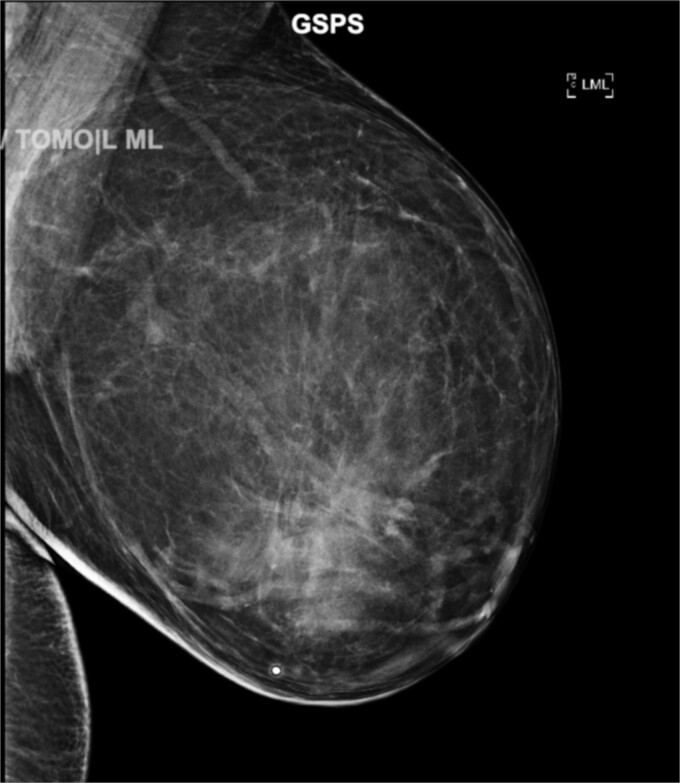
Diagnostic mammogram showing encapsulated fatty mass occupying a majority of the left breast. Parenchymal distortion and asymmetry are seen at the center of the mass. The mass measured 20 cm in diameter and was classified as a BI-RADS category 4 [(A) CC view and (B) MLO view].

A diagnostic bilateral mammogram (Fig. 1), demonstrated a large, encapsulated fatty mass occupying a majority of the left breast. Parenchymal distortion and asymmetry were seen at the center of the mass. The mass measured 20 cm in diameter and was classified as Breast Imaging-Reporting and Data System (BI-RADS) category 4. The right breast showed no evidence of disease or malignancy. A focused left breast ultrasound (US) demonstrated similar findings to the mammogram highlighting a hypoechoic mass at the 12 o’clock position.

**Figure 2:**
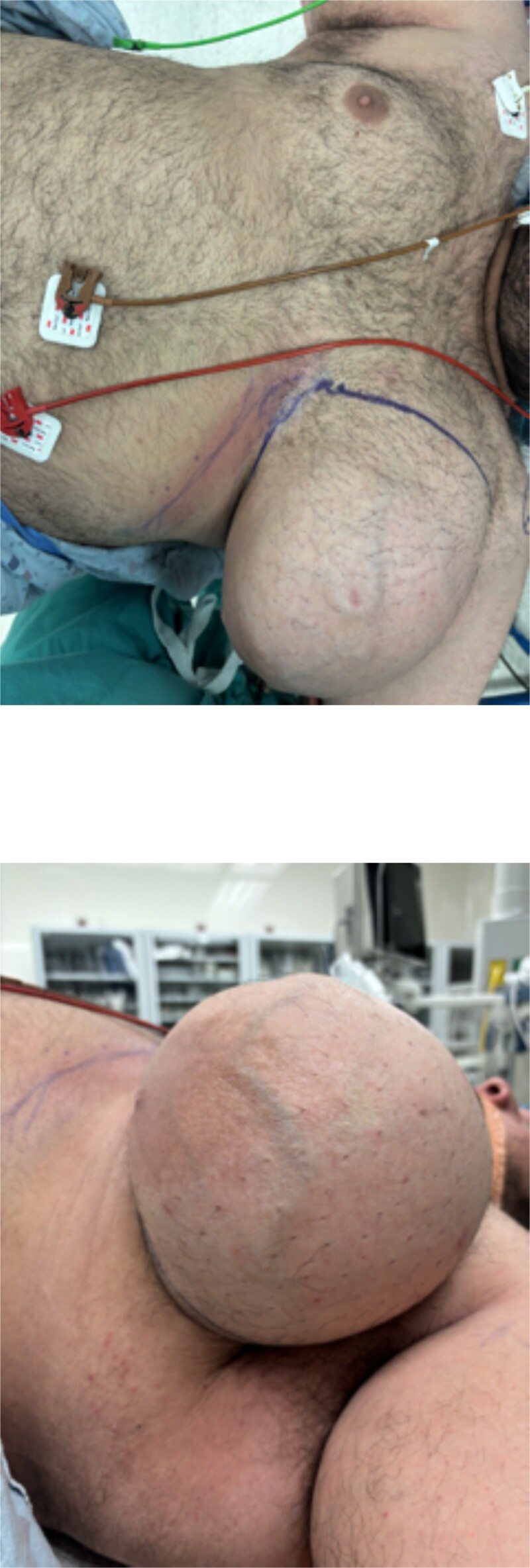
Preoperative gross imaging of the patient’s breast: (a) coronal view and (b) sagittal view.

**Figure 3:**
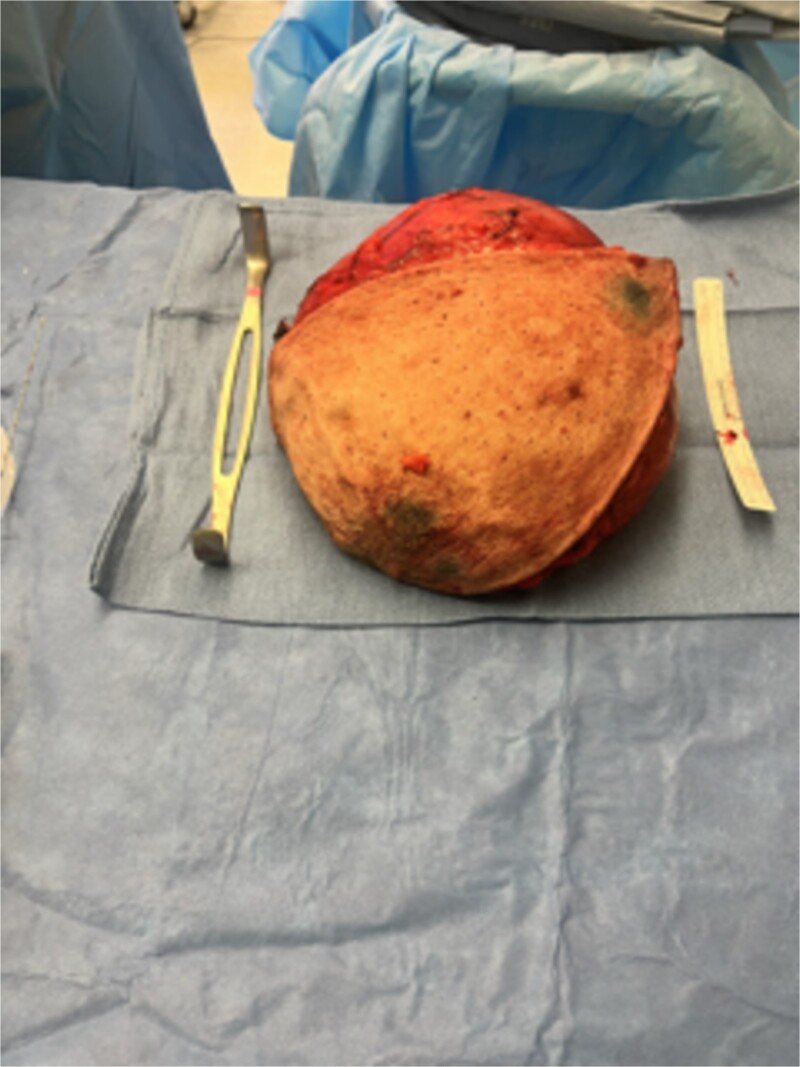
Gross resected breast specimen shows the resected breast mass postoperatively.

Based on the findings of the diagnostic tests, surgical resection of the mass was performed: the patient underwent an intra-operative US-guided needle biopsy and left total mastectomy. Figure 2a and b shows the preoperative gross imaging of the patient’s breast, and Fig. 3 shows the resected breast. The core needle biopsy revealed ‘scant fragments of blood’, and left breast pathology revealed ‘benign nipple, unremarkable skin, and hibernoma with fat necrosis’.

## Discussion

Brown adipose tissue is used for thermoregulation by producing large amounts of heat and is most apparent at birth and in infants. It was once believed that brown fat disappears after puberty; however, studies have shown that it may persist into adulthood [3]. Hibernomas are rarely found in the mammary tissue, with a higher prevalence in females than males [2]. Only one article specifically described a hibernoma of the breast in a male patient, which was treated with mass resection [2]. The low incidence in males and uncommon mass location makes this case report a unique presentation, as it challenges our understanding of the distribution of brown fat tissue and offers a unique diagnosis for the male breast mass.

A large review in 2001 followed 170 hibernoma cases and evaluated their morphological features and behavior: location occurred mostly in the thigh, shoulder, back, and chest, and none were reported in the breast [5]. The hibernomas presented as four morphological variants: typical, myxoid, lipoma-like, and spindle-like. Histologically, hibernomas consist of coarsely multi-vacuolated fat cells with small, central nuclei and no atypia. Myxoid variants demonstrate a loose basophilic matrix, spindle cell variants exhibit features like spindle cell lipomas, and lipoma-like variants contain sparse hibernoma cells [5].

Diagnosing hibernomas can be difficult due to the similarities in presentation to other soft tissue tumors, such as liposarcomas, angiolipomas, lipomas, fibromas, and hibernomas [1, 4, 5]. Imaging modalities, such as US, MRI, CT, and PET scans, may assist in diagnosing hibernomas. Ultrasound evaluation of hibernomas presents as hyperechoic, well-demarcated uniform masses. MRI shows increased signal intensity on both T1 and T2, but may not completely rule out liposarcoma. Color Doppler imaging shows hypervascularity in hibernomas; however, may be nonspecific. PET scans offer diagnostic value due to glucose uptake in brown fat [4]. In most cases, imaging alone is insufficient and pathological evaluation is necessary to establish a diagnosis.

Male breast cancer is uncommon, comprising <1% of breast cancer cases worldwide [6]. While there is significant data and research on female breast cancer, data is limited for male breast cancer. It is thus necessary to consider a broad differential diagnosis when evaluating male breast masses. Benign conditions may include gynecomastia, lipomas, and hibernoma, and the differential must include possible malignancy. The management of breast masses in males is similar to the approach among female patients and can include lumpectomy, simple mastectomy, lymph node dissection, chemotherapy, and hormonal therapy [7]. Expanding the differential diagnosis helps to accurately identify masses, to characterize them as benign or malignant, and to ultimately provide appropriate intervention.

## Conclusion

Hibernomas are rare, slow-growing tumors derived from brown adipose tissue. Hibernomas are rarely found in the mammary tissue, and of those, they are higher in prevalence among females than males. This case report highlights a rare finding of a large 20 cm breast hibernoma in a 31-year-old male. While hibernomas are benign masses, this uncommon diagnosis should be included in the differential when evaluating masses in male patients to prevent delayed diagnosis and treatment.
